# Ulcerative fibroepithetial stromal polyp of the vulva with strong clinical suspicion for vulvar malignancy: A case report and mini‑review of the literature

**DOI:** 10.3892/mi.2025.230

**Published:** 2025-04-01

**Authors:** Efthymia Thanasa, Anna Thanasa, Ioannis-Rafail Antoniou, Emmanouil Xydias, Apostolos Ziogas, Evangelos Kamaretsos, Athanasios Chasiotis, Alexandros Leroutsos, Evangelia Simopoulou, Maria Mousia, Ioannis Thanasas

**Affiliations:** 1 Department of Health Sciences, Medical School, Aristotle University of Thessaloniki, 54124 Thessaloniki, Greece; 2 Department of Obstetrics and Gynecology, General Hospital of Trikala, 42100 Trikala, Greece; 3 Department of Obstetrics and Gynaecology, EmbryoClinic IVF, 55133 Thessaloniki, Greece; 4 Department of Medicine, University of Thessaly, School of Health Sciences, 41334 Larissa, Greece; 5 Department of Pathology, General Hospital of Trikala, 42100 Trikala, Greece

**Keywords:** vulvar fibroepithelial polyp, ulcerative lesions, clinical presentation, wide surgical excision, histological diagnosis

## Abstract

Fibroepithelial stromal polyps of the vulva are rare clinical entities, typically appearing as isolated polypoid stromal lesions, covered by squamous epithelium. The presence of non-traumatic ulcerative lesions on the tumor surface, particularly when the tumor is relatively small, necessitates the differentiation from malignant lesions of the vulva. The present study describes the case of a 44-year-old patient who presented to an outpatient gynecological clinic, reporting as her sole symptom the presence of a mass in the vulval region, which had been increasing in size over the past 12 months. Upon examination and palpation of the external genitalia, a painless pedunculated tumor measuring ~5 cm, originating from the upper third of the right labium majus, covered with normal skin, but bearing ulcerative lesions, was found. An ulcerative fibroepithelial polyp of the vulva was suspected, and surgical treatment with wide excision of the lesion was decided upon. A histological examination of the surgical specimen confirmed the diagnosis. The immunohistochemical analysis of the tumor ruled out malignancy. Following an uneventful post-operative course, the patient was discharged from the clinic the following day. At 6 months post-operatively, no recurrence of the fibroepithelial polyp was found at the site of pedicle resection. The present study, also provides a brief literature review of this rare disease entity following the case presentation, highlighting the necessity of wide surgical excision of vulvar fibroepithelial polyps and their differentiation from vulvar malignancies.

## Introduction

The vulvar lesions described to date exhibit a wide range of morphologies. Among these, some are neoplastic, displaying malignant features and carrying a significant risk of morbidity, while others are non-neoplastic or benign neoplasms (1). Primary vulvar adenocarcinomas are rare occurrences (2). Mesenchymal lesions of the vulva may manifest as locally specific to the vulvar region or as non-specific soft tissue neoplasms, which occur more frequently at sites other than the vulva. Local mesenchymal lesions confined to the lower genital tract include fibroepithelial stromal polyps of the vulva, cellular angiofibroma, angiomyofibroblastoma, superficial angiomyxoma and aggressive angiomyxoma (3).

Fibroepithelial stromal polyps of the female genital tract were initially described as a distinct clinical entity by Norris and Taylor (4) in the early 1960s. This lesion is benign and typically occurs in women of reproductive age, more frequently in the vagina than in the cervix and vulva (5). Fibroepithelial stromal polyps of the vulva typically present as solitary polypoid stromal lesions covered by normal squamous epithelium (6). Their size is usually not >5 cm, although in rare cases, they may reach sizes of up to 20 cm and may weigh >1 kg (7). The pathogenesis of vulvar fibroepithelial polyps is largely unknown; however, hormonal stimulation and chronic inflammation are considered major predisposing risk factors (8).

The present study describes the case of a patient of vulvar fibroepithelial polyp, emphasizing the rarity of this clinical entity. Additionally, it addresses the clinical, imaging and histological diagnostic approach to these lesions, highlighting the necessity of surgical resection and the importance of differentiating them from malignant vulvar lesions.

## Case report

A 44-year-old female patient of reproductive age presented at the Outpatient Gynecological Clinic of the General Hospital of Trikala, Trikala, Greece. She reported that the sole reason for her visit was the presence of a small tumor on her vulva, which was not associated with neither pain nor tenderness. The patient only reported experiencing mild discomfort in her external genitalia. No history of trauma or friction injury to the vulvar area was reported, and there were no clinical signs of vulvar infection or inflammation. The patient could not recall when the polyp first appeared; however, she had stated that over the past year, she noted an increase in its size, prompting her to consult a gynecologist. Her menstrual cycle was reported as normal, she had a normal hormonal status, typical of a pre-menopausal woman of her age and no abnormal findings were noted from the last Pap smear test, which had been conducted 2 years prior as part of her gynecological screening. As regards her obstetric history, she had three lower-segment cesarean deliveries. Additionally, the patient reported a medical history of hypothyroidism, which was effectively controlled with appropriate medical treatment. She reported no other chronic or metabolic disorders.

**Figure 1:**
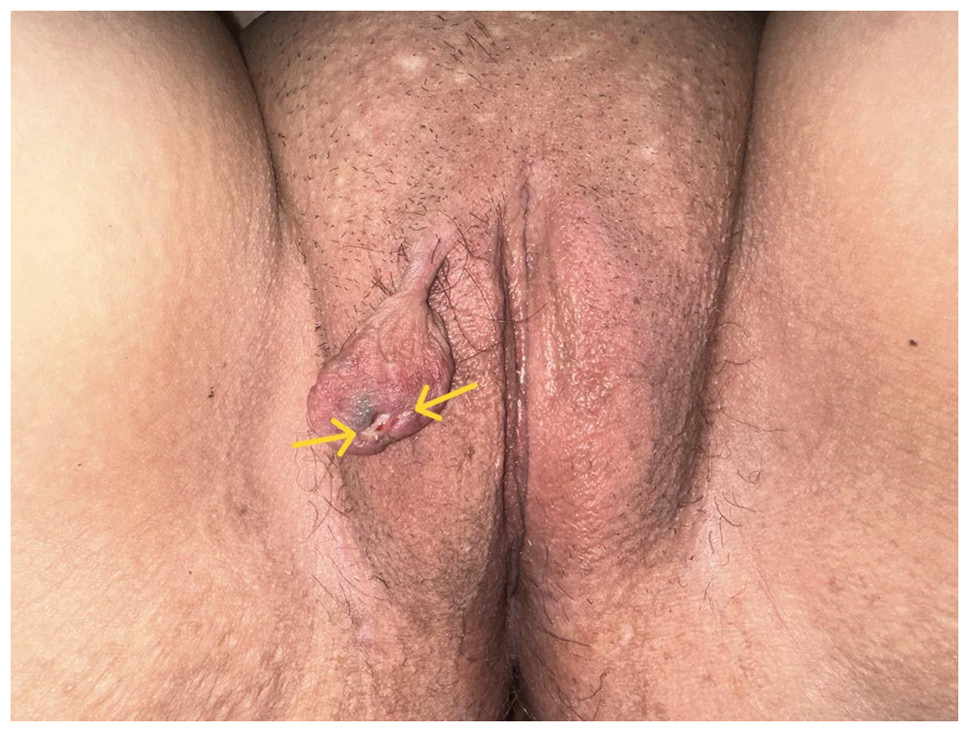
Fibroepithelial stromal polyp of the vulva: The presence of non-traumatic ulcerative lesions on the surface of the tumor (indicated by yellow arrows) necessitates its differentiation from vulvar malignancy.

Upon a clinical examination, a painless pedunculated tumor was noted at the external genitalia, with a maximal diameter of ~5 cm including the pedicle. The tumor originated from the upper section of the anterior surface of the right labium majus, near the clitoris. It exhibited density on palpation and was covered with normal skin, displaying ulcerative lesions (Fig. 1). No abnormal findings were observed during a clinical and transvaginal ultrasound examination of the internal genitalia. The Pap smear test conducted at that time as part of preventive screening yielded normal results. The levels of inflammatory markers and tumor markers were within normal laboratory values (Table I).

Based on the clinical findings, a fibroepithelial polyp of the vulva was suspected and the patient was scheduled for surgery after obtaining her informed consent. In the operating room, under spinal anesthesia, the wide excision of the pedunculated tumor was performed, followed by suturing of the surgical wound.

**Figure 2:**
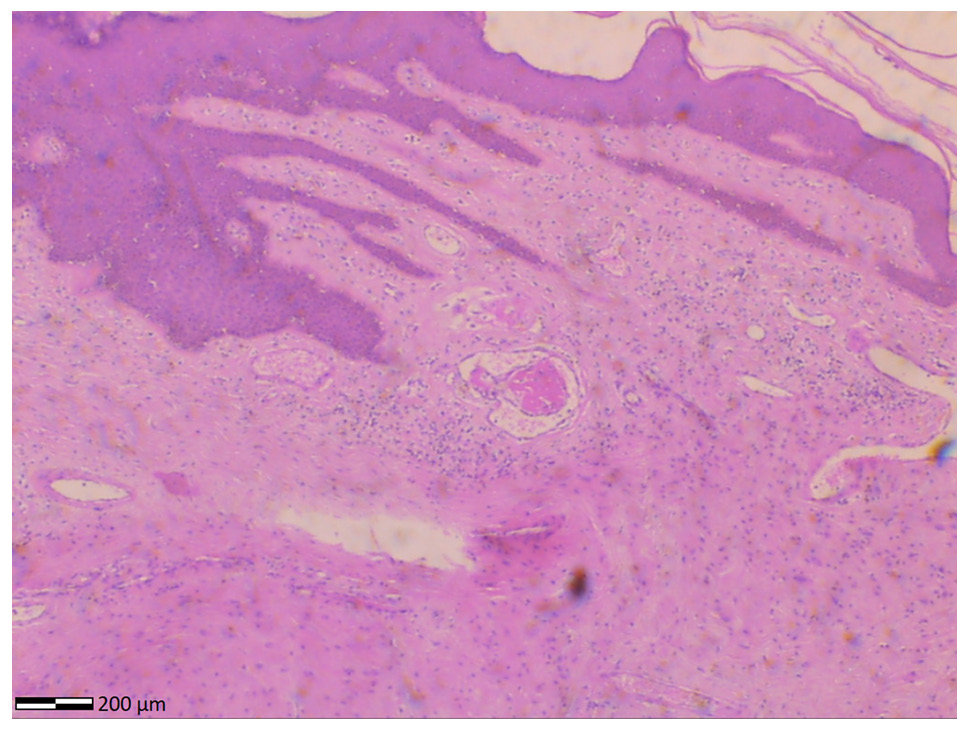
Histological image of fibroepithelial stromal polyp of the vulva: hypocellular stroma without atypia, epidermis with hyperkeratosis and several blood vessels are seen (hematoxylin and eosin staining; magnification, x4).

The collected surgical specimens were sent to the Histopathology Laboratory of the Hospital (Anatomic Pathology Laboratory of the General Hospital of Trikala) for further assessment. The pathological examination of the surgical specimen macroscopically revealed a polypoidal stromal lesion extending to the epidermis, with the presence of several blood vessels. Microscopic analysis was performed utilizing hematoxylin and eosin staining. As per the Laboratory's routine protocol, specimens were embedded in paraffin cubes and 5-mcm-thick sections were obtained for analysis. A buffered, 10% formalin solution was utilized as a fixative medium, for 36 h at room temperature. Hematoxylin and eosin staining 0.5% alcohol (Diachel A.E.) was applied for 12 min at room temperature. All microscopic examinations were performed using a LEICA DM2000 optical microscope (Leica Microsystems GmbH). The microscopic examination revealed spindle-shaped and stellate cells without atypia or mitoses, collagen deposition, congestion and localized thrombosis of the stromal vessels. In addition, epidermis with hyperplasia and hyperkeratosis was observed and no dysplastic epithelial lesions were observed (Fig. 2).

**Figure 3:**
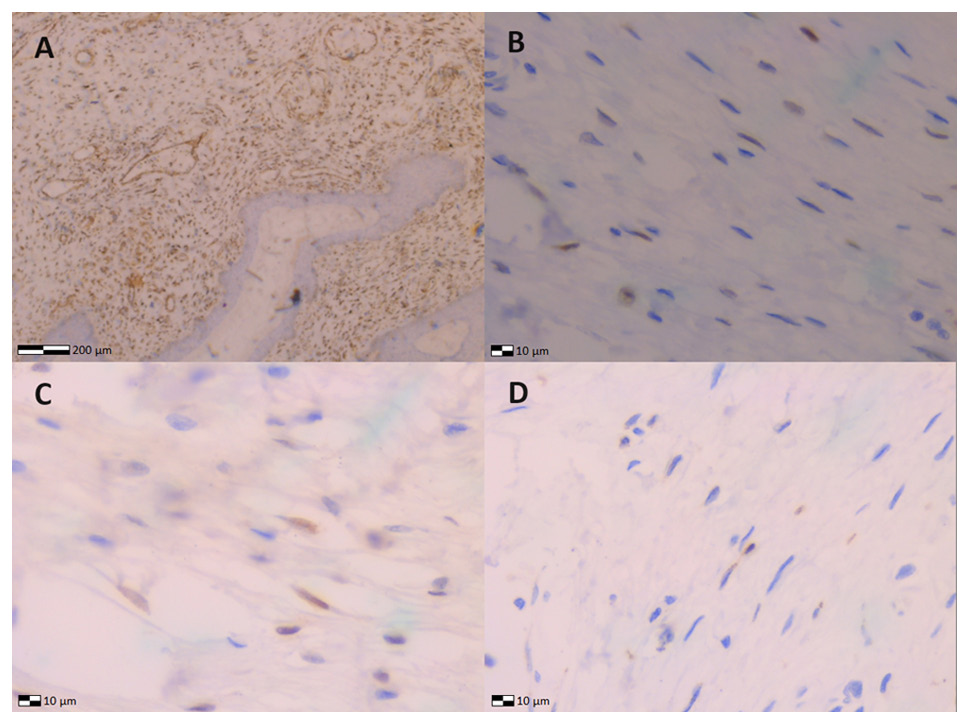
Immunohistochemical images of fibroepithelial stromal polyp of the vulva illustrating: (A) Vimentin positivity (magnification, x4), (B) estrogen receptor positivity (magnification, x4), (C) progesterone receptor positivity (magnification, x4), and (D) Ki67 positivity (magnification, x4).

The specimens were also subjected to immunohistochemical analysis. Paraffin-embedded sections 4µm thick were collected and dewaxed for 40 min at 70˚C. Subsequently, they were placed sequentially in BOND™ Dewax solution, 100% v/v ethanol solution and BOND™ wash solution. Heat-induced epitope retrieval (HIER) was performed via the use of BOND™ Epitope Retrieval ER1 Solution, (pH 7) for estrogen receptor (ER), progesterone receptor (PR), Vimentin (VIM) and Ki67 for 20 min at 100˚C. The block peroxide kit (Bond; Leica Biosystems) was used for 5 min. Antibody dilution was performed with a proprietary Leica (Bond; Leica Biosystems) solution as follows: Dilution for ER (M3643; Agilent Technologies, Inc.) was 1:40, for PR (M3569; Agilent Technologies, Inc.) was 1:100, for VIM (M0725; Agilent Technologies, Inc.) was 1:100 and for Ki67 (F0788; Agilent Technologies, Inc.) was 1:20. All antibodies were incubated for a period of 30 min in total. In particular, the post-primary kit (Bond; Leica Biosystems) was used for a duration of 10 min at an incubation temperature of 100˚C. Subsequently, a secondary detection kit polymer (Bond; Leica Biosystems) was used for a duration of 10 min and the DAB kit (Bond; Leica Biosystems) for 10 min to facilitate visualization. Hematoxylin was applied for 5 min as a counterstain at room temperature and the sections were dehydrated, mounted and coverslipped. The resulting slides were examined under the same LEICA DM2000 optical microscope (Leica Microsystems GmbH). The specimen exhibited positivity for vimentin (VIM), estrogen receptors (ER), progesterone receptors (PR) and Ki67 (Fig. 3).

The post-operative course of the patient was uneventful, and the patient was discharged from the clinic on the first post-operative day. At 6 months post-operatively, no recurrence of the fibroepithelial polyp was observed at its extraction site from the vulva. The patient was advised to follow-up with annual visits to the gynecological clinic at the General Hospital of Trikala.

## Discussion

The diagnosis of vulvar fibroepithelial polyps presents significant challenges. Ultrasound findings, magnetic resonance imaging findings and detailed histological analysis need to be combined with the clinical presentation of the tumor, which is typically non-specific (9). Small fibroepithelial polyps of the vulva (<5 cm) are often asymptomatic and are incidentally discovered during gynecological examinations. In other cases, patients may report their presence when seeking medical attention at a later stage. In rare cases of large fibroepithelial polyps (>5 cm), symptoms are more likely to occur, with bleeding being the most frequent clinical manifestation. Additionally, ulcerative lesions and signs of inflammation, often accompanied by varying degrees of secondary infection, may be associated with large vulvar fibroepithelial polyps (10,11). A single case report of a patient with sepsis attributed to a huge ulcerative and inflammatory fibroepithelial polyp of the vulva has been documented in the international literature (12). In the patient in the present study, the small size of the polyp (<5 cm) with the presence of a thick pedicle without signs of vascular obstruction or torsion (Fig. 1) did not explain the presence of ulcerative lesions due to insufficient blood supply. Therefore, considering the absence of trauma to the vulvar region, the presence of ulcerative lesions on the surface of the tumor raised strong suspicion regarding the possibility of malignancy. Benign vulvar fibroepithelial polyps can often be mistaken for malignant tumors due to their diverse morphological features (13). Malignant vulvar lesions that should be included in the differential diagnosis of fibroepithelial polyps include aggressive angiomyxoma, angiomyofibroblastoma, sarcoma, superficial cervical and vaginal myofibroblastoma, cellular angiofibroma, perineurioma, botryoid rhabdomyosarcoma, and squamous cell carcinoma (8,14).

Contemporary imaging modalities can markedly aid in the pre-operative diagnosis of vulvar fibroepithelial polyps, particularly when they are of substantial size. An ultrasound is considered a first-line imaging modality, being more accessible and cost-effective than computed tomography and magnetic resonance imaging (5). An ultrasound can detect the presence of soft tissue within the mass and, depending on the location of the fibroepithelial polyp in the vulvar region, and it can exclude the presence of a hernia containing omentum or intestine (11). Doppler ultrasonography may reveal a central hypoechoic mass of varying dimensions with increased peripheral vascularity (15). Additionally, findings from magnetic resonance imaging, such as stromal hypodense areas on T2-weighted scans and linear hyperdense areas on T1 images, are suggestive of vulvar fibroepithelial polyps (16). In 2019, Yoo *et al* (14) reported that contrast-enhanced diffusion-weighted magnetic resonance imaging can aid in differentiating vulvar fibroepithelial polyps from other vulvovaginal stromal tumors. In the patient described herein, given the small size of the tumor, ultrasound and magnetic resonance imaging were not deemed necessary as part of the preoperative evaluation. Based on the clinical findings, it was decided to proceed with the wide surgical excision of the lesion followed by histological examination of the tumor.

Accurate histological analysis of vulvar fibroepithelial polyps is crucial for diagnosis. Microscopic examination of the tumor is essential for distinguishing vulvar fibroepithelial polyps from malignant vulvar lesions (17). Histologically, vulvar fibroepithelial polyps exhibit a cellular stromal component with a central vascular core, covered by overlying benign squamous epithelium. The hallmark of vulvar fibroepithelial polyps is the presence of stellate and multinucleated stromal cells, typically located at the epithelium-stroma interface. These cells express estrogen and progesterone receptors, react to desmin, and occasionally to smooth muscle fiber actin (18,19). In the patient in the present study, a histological examination ruled out the presence of vulvar malignancy. The immunohistochemical analysis of the tumor was performed to confirm its differentiation from malignant vulvar lesions. In the case of the patient described herein, the importance of wide surgical excision of the lesion and the necessity of accurate histological diagnosis are emphasized, as the risk of local recurrence varies for these tumors (18).

Total surgical resection is the preferred treatment option for vulvar fibroepithelial polyps, particularly for those that are large in size. Cryotherapy or cauterization may be considered ideal treatment modalities for small polyps (20,21). Timely and proper surgical treatment, accompanied by definitive histological diagnosis, is considered to significantly contribute to the radical management of vulvar fibroepithelial polyps and to the management of anxiety in these patients (22). Although not common, local recurrence can be observed, particularly in cases where the polyp resection is incomplete (23). In the patient in the present study, there was no evidence of local recurrence at 6 months post-operatively. To ensure the early detection of potential future recurrence, the patient was advised to undergo regular follow-up with annual visits to the outpatient gynecological clinic of the General Hospital of Trikala.

In conclusion, fibroepithelial stromal polyps of the vulva are rare benign mesenchymal tumors that typically occur in women of reproductive age. Following the wide surgical excision of the vulvar lesion, confirmation of the diagnosis through meticulous microscopic histological examination of the surgical specimen is necessary in all cases. Furthermore, the presence of non-traumatic ulcerative lesions on the surface of the polyp, even in cases where tumors are small in size, should raise clinical suspicion of vulvar malignancy, prompting the implementation of immunohistochemical assessment of the tumor to definitively differentiate it from vulvar malignancies.

## Figures and Tables

**Table I:** The results of laboratory tests that were performed during the pre-operative assessment of the patient.

Laboratory tests	Preoperative values	Laboratory reference values
Ht	34.1%	37.7-49.7%
Hb	10.6 g/dl	11.8-17.8 g/dl
PLT	331x10^3^/ml	150-350x10^3^/ml
WBC	6.76x40^3^/ml	4-10.8x10^3^/ml
NEUT	49.8%	40-75%
APTT	27.4 sec	24.0-35.0 sec
INR	1.05	0.8-1.2
CRP	0.1 mg/dl	0.5 mg/dl
Glu	88 mg/dl	75-115 mg/dl
Cr	0.83 mg/dl	0.40-1.10 mg/dl
CEA	2.26 ng/ml	<5 ng/ml
CA125	21.3 U/ml	≤35 U/ml
CA15-3	17.6 U/ml	0.0-31.3 U/ml
CA15-9	14.9 U/ml	0.0-37 U/ml

Ht, hematocrit; Hb, hemoglobin; PLT, platelets; WBC, white blood cells; NEUT, neutral; APTT, activated partial thromboplastin time; INR, international normalized ratio; CRP, C-reactive protein; U, urea; Glu, glucose; Cr, creatinine; CEA, carcinoembryonic antigen; CA125, cancer antigen 125; CA15-3, cancer antigen 15-3; CA15-9, cancer antigen 19-9.

## Data Availability

The data used in the current study are available from the corresponding author upon reasonable request.
